# Supplementary material for: Significant Interplay Between Lipids, Cytokines, Chemokines, Growth Factors, and Blood Cells in an Outpatient Cohort
Source: Int J Mol Sci. 2025 Aug 11;26(16):7746. doi: 10.3390/ijms26167746 (PMC12387115; doi:10.3390/ijms26167746)
Supplement: Supplementary file 1 [file ijms-26-07746-s001.zip › 20250809_Suppl Tables 1 _2.pdf]

Suppl.Table S1

Biomarker	Cytokine	Valid	Spearman	p-value	Benjamini-Hochberg significance	Benjamini-Hochberg P-value
Age	Flt3L	164	0.299	0.000	significant	0.005
Age	TGF-alpha	164	-0.206	0.008	significant	0.065
Age	CCL20	164	-0.214	0.006	significant	0.053
Age	ADA	164	-0.220	0.005	significant	0.047
Age	LTA	164	-0.247	0.001	significant	0.026
Age	S100A12	164	-0.253	0.001	significant	0.022
Age	TNFRSF9	164	-0.266	0.001	significant	0.013
Age	IL-18R1	164	-0.315	0.000	significant	0.003
Albumin	DNER	164	0.287	0.000	significant	0.010
Albumin	S100A12	164	0.253	0.001	significant	0.027
Albumin	PLAU	164	0.232	0.003	significant	0.043
Albumin	TNFSF12	164	0.221	0.004	significant	0.057
Albumin	TNFSF10	164	0.218	0.005	significant	0.060
Albumin	Beta-NGF	164	-0.194	0.013	significant	0.086
Albumin	CD5	164	-0.212	0.007	significant	0.064
Albumin	TNF	164	-0.224	0.004	significant	0.052
Albumin	CSF-1	164	-0.296	0.000	significant	0.009
ApoA1	TNFSF10	164	-0.190	0.015	significant	0.086
ApoA1	CCL3	164	-0.194	0.013	significant	0.086
ApoA1	TNFRSF9	164	-0.194	0.013	significant	0.086
ApoA1	CD6	164	-0.197	0.011	significant	0.084
ApoA1	CCL19	164	-0.205	0.008	significant	0.072
ApoA1	TNFSF11	164	-0.217	0.005	significant	0.060
ApoA1	CCL7	164	-0.226	0.004	significant	0.050
ApoA1	S100A12	164	-0.235	0.002	significant	0.040
ApoA1	PLAU	164	-0.267	0.001	significant	0.018
ApoB	CCL3	164	0.290	0.000	significant	0.009

ApoB	IL7	164	0.283	0.000	significant	0.011
ApoB	COL18A1	164	0.191	0.015	significant	0.086
ApoB	IL-18R1	164	0.189	0.015	significant	0.086
ApoB	TNFSF11	164	0.189	0.015	significant	0.086
ApoB	IL-15RA	164	0.184	0.018	significant	0.094
ApoB	TNFSF10	164	0.182	0.019	significant	0.096
BMI	IL6	164	0.401	0.000	significant	0.000
BMI	CCL7	164	0.325	0.000	significant	0.002
BMI	HGF	164	0.312	0.000	significant	0.003
BMI	CCL19	164	0.305	0.000	significant	0.004
BMI	CDCP1	164	0.252	0.001	significant	0.023
BMI	FGF-21	164	0.244	0.002	significant	0.026
BMI	TNFSF11	164	0.241	0.002	significant	0.028
BMI	CSF-1	164	0.227	0.003	significant	0.040
BMI	IL-12B	164	0.224	0.004	significant	0.042
BMI	CCL3	164	0.217	0.005	significant	0.051
BMI	CCL4	164	0.203	0.009	significant	0.072
BMI	OSM	164	0.192	0.014	significant	0.090
BMI	TNFSF10	164	0.190	0.015	significant	0.092
BMI	FGF-19	164	-0.278	0.000	significant	0.009
Creatinine	IL-17C	164	0.314	0.000	significant	0.003
Creatinine	FGF-19	164	0.306	0.000	significant	0.004
Creatinine	MMP-10	164	0.241	0.002	significant	0.028
Creatinine	CCL25	164	0.241	0.002	significant	0.028
Creatinine	TNFSF12	164	0.239	0.002	significant	0.029
Creatinine	CX3CL1	164	0.227	0.004	significant	0.040
Creatinine	TNFRSF9	164	0.214	0.006	significant	0.053
Creatinine	TGF-alpha	164	0.194	0.013	significant	0.087
Creatinine	CCL11	164	0.192	0.014	significant	0.090
Creatinine	TNFSF10	164	0.190	0.015	significant	0.092
eGFR	CCL7	164	0.198	0.011	significant	0.080
eGFR	IL6	164	0.195	0.012	significant	0.085
eGFR	FGF-19	164	-0.215	0.006	significant	0.052

Gender	CASP-8	164	-0.217	0.005	significant	0.051
Gender	HGF	164	-0.222	0.004	significant	0.045
Gender	IL-18R1	164	-0.231	0.003	significant	0.036
Gender	TNFSF11	164	-0.233	0.003	significant	0.035
Gender	ADA	164	-0.249	0.001	significant	0.025
Gender	CCL25	164	-0.250	0.001	significant	0.024
Gender	TNFRSF9	164	-0.261	0.001	significant	0.017
Gender	S100A12	164	-0.269	0.000	significant	0.012
Gender	CCL2	164	-0.278	0.000	significant	0.009
Gender	TNFSF12	164	-0.292	0.000	significant	0.006
Gender	PLAU	164	-0.294	0.000	significant	0.005
Gender	CCL11	164	-0.300	0.000	significant	0.005
Gender	DNER	164	-0.321	0.000	significant	0.003
Gender	IL-1 alpha	164	-0.331	0.000	significant	0.002
Gender	TNFSF10	164	-0.403	0.000	significant	0.000
HDL	TNFRSF9	164	-0.190	0.015	significant	0.086
HDL	IL6	164	-0.190	0.015	significant	0.086
HDL	HGF	164	-0.192	0.014	significant	0.086
HDL	FGF-21	164	-0.195	0.012	significant	0.085
HDL	PLAU	164	-0.201	0.010	significant	0.081
HDL	S100A12	164	-0.211	0.007	significant	0.064
HDL	CDCP1	164	-0.213	0.006	significant	0.064
HDL	TNFSF10	164	-0.217	0.005	significant	0.060
HDL	TNFSF11	164	-0.229	0.003	significant	0.048
HDL	CCL7	164	-0.240	0.002	significant	0.034
HDL	CCL19	164	-0.244	0.002	significant	0.031
HDL	CCL3	164	-0.286	0.000	significant	0.010
LDL	TNFSF10	164	0.198	0.011	significant	0.083
LDL	CDCP1	164	0.196	0.012	significant	0.085
Non-HDL	CCL3	164	0.248	0.001	significant	0.031
Non-HDL	CDCP1	164	0.247	0.001	significant	0.031
Non-HDL	TNFSF10	164	0.201	0.010	significant	0.081

Non-HDL	HGF	164	0.184	0.018	significant	0.094
Non-HDL	TNFSF11	164	0.183	0.019	significant	0.095
Remnants	FGF-21	164	0.245	0.002	significant	0.031
Remnants	CCL3	164	0.244	0.002	significant	0.031
Remnants	COL18A1	164	0.237	0.002	significant	0.038
Remnants	CDCP1	164	0.226	0.004	significant	0.050
Remnants	IL-10RB	164	0.215	0.006	significant	0.062
Remnants	IL6	164	0.207	0.008	significant	0.070
Remnants	CASP-8	164	0.199	0.011	significant	0.083
Remnants	SULT1A1	164	0.184	0.018	significant	0.094
Triglycerides	FGF-21	164	0.245	0.002	significant	0.031
Triglycerides	CCL3	164	0.245	0.002	significant	0.031
Triglycerides	COL18A1	164	0.237	0.002	significant	0.038
Triglycerides	CGCP1	164	0.227	0.003	significant	0.049
Triglycerides	IL-10RB	164	0.216	0.006	significant	0.061
Triglycerides	IL6	164	0.207	0.008	significant	0.070
Triglycerides	CASP-8	164	0.199	0.011	significant	0.083
Triglycerides	SULT1A1	164	0.184	0.018	significant	0.094
Weight	IL6	164	0.342	0.000	significant	0.002
Weight	TNFSF11	164	0.327	0.000	significant	0.002
Weight	TNFSF10	164	0.327	0.000	significant	0.002
Weight	HGF	164	0.308	0.000	significant	0.004
Weight	CDCP1	164	0.293	0.000	significant	0.005
Weight	CCL19	164	0.289	0.000	significant	0.006
Weight	IL-18R1	164	0.272	0.000	significant	0.011
Weight	IL-10RB	164	0.269	0.000	significant	0.012
Weight	FGF-21	164	0.234	0.003	significant	0.034
Weight	PLAU	164	0.230	0.003	significant	0.037
Weight	CCL7	164	0.215	0.006	significant	0.052
Weight	CCL3	164	0.211	0.007	significant	0.057
Weight	CCL20	164	0.204	0.009	significant	0.068
Weight	CD5	164	0.198	0.011	significant	0.080
Weight	CXCL11	164	-0.210	0.007	significant	0.059

Weight	CCL28	164	-0.228	0.003	significant	0.039

Supplementary Table S1. Significant associations between biomarkers [Albumin (g/L)], [Apolipoprotein A1 (ApoA1; g/L)], [Apolipoprotein B (Apo B; g/L), [HDL (high-density lipoprotein cholesterol; mmol/L)], [Triglycerides (mmol/L)], [Non-HDL (high-density lipoprotein cholesterol; mmol/L)], [LDL (low density lipoprotein cholesterol; mmol/L)], [Remnants (Non-HDL – LDL; mmol/L)], creatinine (micromole/L), and [$eGFR_{creatinine}$ (estimated glomerular filtration rate; mL/min/1.73 square meters)], versus cytokines.

Suppl. Table S2

Biomarker	Hematology	Valid	Spearman	p-value	Benjamini-Hochberg significance	Benjamini-Hochberg P-value
Age	MCHC	164	-0.168	0.031	significant	0.070
Albumin	Hemoglobin	164	0.438	0.000	significant	0.000
Albumin	EVF	164	0.419	0.000	significant	0.000
Albumin	Ery	164	0.331	0.000	significant	0.000
Albumin	MCHC	164	0.285	0.000	significant	0.001
Albumin	MCH	164	0.162	0.037	significant	0.080
ApoA1	MCV	164	0.194	0.012	significant	0.033
ApoB	Ery	164	0.314	0.000	significant	0.000
ApoB	TPK	164	0.223	0.004	significant	0.016
ApoB	EVF	164	0.214	0.006	significant	0.021
ApoB	Hemoglobin	164	0.206	0.008	significant	0.024
ApoB	MCV	164	-0.217	0.005	significant	0.019
ApoB	MCH	164	-0.240	0.002	significant	0.008
BMI	WBC	164	0.322	0.000	significant	0.000
BMI	Neutrophils	164	0.253	0.001	significant	0.005
BMI	TPK	164	0.250	0.001	significant	0.005
BMI	Ery	164	0.181	0.020	significant	0.049
BMI	MCV	164	-0.181	0.020	significant	0.049
Creatinine	EVF	164	0.475	0.000	significant	0.000
Creatinine	Hemoglobin	164	0.472	0.000	significant	0.000
Creatinine	Ery	164	0.439	0.000	significant	0.000
Creatinine	MCHC	164	0.179	0.022	significant	0.051
Gender	MCHC	164	-0.407	0.000	significant	0.000
Gender	Ery	164	-0.648	0.000	significant	0.000
Gender	EVF	164	-0.692	0.000	significant	0.000
Gender	Hemoglobin	164	-0.717	0.000	significant	0.000

HDL	MCV	164	0.198	0.011	significant	0.030
HDL	MCHC	164	-0.164	0.034	significant	0.076
HDL	Ery	164	-0.180	0.021	significant	0.049
LDL	Ery	164	0.229	0.003	significant	0.012
LDL	MCH	164	-0.191	0.014	significant	0.036
Non-HDL	Ery	164	0.303	0.000	significant	0.001
Non-HDL	TPK	164	0.209	0.007	significant	0.023
Non-HDL	Hemoglobin	164	0.206	0.008	significant	0.024
Non-HDL	EVF	164	0.205	0.008	significant	0.024
Non-HDL	MCV	164	-0.206	0.008	significant	0.024
Non-HDL	MCH	164	-0.237	0.002	significant	0.009
Remnants	Ery	164	0.318	0.000	significant	0.000
Remnants	WBC	164	0.290	0.000	significant	0.001
Remnants	TPK	164	0.286	0.000	significant	0.001
Remnants	Hemoglobin	164	0.271	0.000	significant	0.002
Remnants	EVF	164	0.261	0.001	significant	0.003
Remnants	Neutrophils	164	0.195	0.012	significant	0.032
Remnants	MCV	164	-0.211	0.007	significant	0.022
Tot. Cholest	Ery	164	0.222	0.004	significant	0.016
Tot. Cholest	TPK	164	0.163	0.037	significant	0.080
Tot. Cholest	MCH	164	-0.193	0.013	significant	0.033
Triglycerides	Ery	164	0.316	0.000	significant	0.000
Triglycerides	WBC	164	0.291	0.000	significant	0.001
Triglycerides	TPK	164	0.287	0.000	significant	0.001
Triglycerides	Hemoglobin	164	0.270	0.000	significant	0.002
Triglycerides	EVF	164	0.260	0.001	significant	0.004
Triglycerides	Neutrophils	164	0.196	0.012	significant	0.032
Triglycerides	MCV	164	-0.211	0.006	significant	0.022
Weight	Ery	164	0.403	0.000	significant	0.000

Weight	Hemoglobin	164	0.380	0.000	significant	0.000
Weight	EVF	164	0.371	0.000	significant	0.000
Weight	WBC	164	0.266	0.001	significant	0.003
Weight	Neutrophils	164	0.206	0.008	significant	0.024
Weight	MCHC	164	0.186	0.017	significant	0.042
Weight	TPK	164	0.178	0.022	significant	0.051
Weight	MCV	164	-0.210	0.007	significant	0.023

Supplementary Table S2. Significant associations between:

Biomarkers: Age (Years), albumin (g/L), ApoA1 [Apolipoprotein A1 (g/L)], ApoB [Apolipoprotein B), BMI [body mass index (kg/m^2)], creatinine (micrmol/L), [HDL (high-density lipoprotein cholesterol; mmol/L)], triglyc [Triglycerides (mmol/L)], [Non-HDL (high-density lipoprotein cholesterol; mmol/L)], [LDL (low density lipoprotein cholesterol; mmol/L)], [Remnants (Non-HDL – LDL; mmol/L)], and Tot. Cholest [total cholesterol (mmol/L)].

Hematological data: Hemoglobin (g/L), ery [erythrocyte count (red blood cells/L)], EVF [(erythrocyte volume fraction)], MCV [(mean corpuscular volume; microm^3)], MCHC [(mean corpuscular hemoglobin concentration; g/L)], MCH [(Mean Corpuscular Hemoglobin) (pg/cell)], TPK [platelets (10^9/L)], WBC [(white blood cells; 10^9/L)], and Neutrophils (10^9/L).
